# Carbon monoxide-loaded red blood cells ameliorate metabolic dysfunction-associated steatohepatitis progression via enhancing AMP-activated protein kinase activity and inhibiting Kupffer cell activation

**DOI:** 10.1016/j.redox.2024.103314

**Published:** 2024-08-17

**Authors:** Hiroki Yanagisawa, Hitoshi Maeda, Isamu Noguchi, Motohiko Tanaka, Naoki Wada, Taisei Nagasaki, Kazuki Kobayashi, Gai Kanazawa, Kazuaki Taguchi, Victor Tuan Giam Chuang, Hiromi Sakai, Hiroyuki Nakashima, Manabu Kinoshita, Hiroaki Kitagishi, Yasuko Iwakiri, Yutaka Sasaki, Yasuhito Tanaka, Masaki Otagiri, Hiroshi Watanabe, Toru Maruyama

**Affiliations:** a Department of Biopharmaceutics, Graduate School of Pharmaceutical Sciences, Kumamoto University, Kumamoto, Japan; b Department of Gastroenterology and Hepatology, Graduate School of Medical Sciences, Kumamoto University, Kumamoto, Japan; c Department of Gastroenterology and Hepatology, Saiseikai Kumamoto Hospital, Kumamoto, Japan; d Division of Pharmacodynamics, Faculty of Pharmacy, Keio University, Tokyo, Japan; e Pharmacy Discipline, Curtin Medical School, Faculty of Health Sciences, Curtin University, GPO Box U1987, Perth, 6845, Western Australia, Australia; f Department of Chemistry, Nara Medical University, Nara, Japan; g Department of Immunology and Microbiology, National Defense Medical College, Tokorozawa, Saitama, Japan; h Department of Molecular Chemistry and Biochemistry, Doshisha University, Kyotanabe, Kyoto, 610-0321, Japan; i Section of Digestive Diseases, Yale University School of Medicine, New Haven, CT, 06510, United States; j Faculty of Pharmaceutical Sciences and DDS Research Institute, Sojo University, Kumamoto, Japan; k Department of Clinical Pharmacy and Therapeutics, Graduate School of Pharmaceutical Sciences, Kumamoto University, Kumamoto, Japan

**Keywords:** Metabolic dysfunction-associated steatohepatitis (MASH), Carbon monoxide (CO), Heme oxygenase-1 (HO-1), Inflammation, Fibrosis, Liver regeneration, AMP-Activated protein kinase (AMPK)

## Abstract

Metabolic dysfunction-associated steatohepatitis (MASH) is a progressive form of nonalcoholic fatty liver disease characterised by fat accumulation, inflammation, oxidative stress, fibrosis, and impaired liver regeneration. In this study, we found that heme oxygenase-1 (HO-1) is induced in both MASH patients and in a MASH mouse model. Further, hepatic carbon monoxide (CO) levels in MASH model mice were >2-fold higher than in healthy mice, suggesting that liver HO-1 is activated as MASH progresses. Based on these findings, we used CO-loaded red blood cells (CO-RBCs) as a CO donor in the liver, and evaluated their therapeutic effect in methionine-choline deficient diet (MCDD)-induced and high-fat-diet (HFD)-induced MASH model mice. Intravenously administered CO-RBCs effectively delivered CO to the MASH liver, where they prevented fat accumulation by promoting fatty acid oxidation via AMP-activated protein kinase (AMPK) activation and peroxisome proliferator-activated receptor induction. They also markedly suppressed Kupffer cell activation and their corresponding anti-inflammatory and antioxidative stress activities in MASH mice. CO-RBCs also helped to restore liver regeneration in mice with HFD-induced MASH by activating AMPK. We confirmed the underlying mechanisms by performing *in vitro* experiments in RAW264.7 cells and palmitate-stimulated HepG2 cells. Taken together, CO-RBCs show potential as a promising cellular treatment for MASH.

## Highlights

• Hepatic HO-1 expression and CO levels increase in MASH patients and model mice.
• Intravenous CO-RBCs delivery has preventive and therapeutic effects in MASH mice.
• CO-RBCs inhibit hepatocyte lipid accumulation by inducing AMPK and PPARα.
• CO-RBCs restore defective liver regeneration in MASH model mice.
• CO-RBCs have anti-oxidative and anti-inflammatory effects on Kupffer cells.

## Graphical abstract

**Figure:**
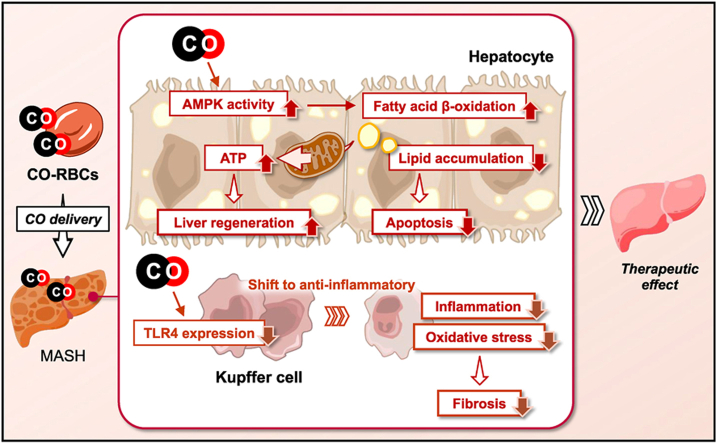

## 1 Introduction

Metabolic dysfunction-associated steatohepatitis (MASH) is a progressive fatty liver disease for which there is no effective treatment. Unfortunately, the prevalence of MASH is on the rise, particularly in developed countries [1,2], and in the advanced disease stages, it can lead to liver cirrhosis and hepatocellular carcinoma [3,4].

The "multiple parallel hits hypothesis” postulates that the interaction of multiple factors, such as metabolic network disruption due to fat accumulation, cytokine induction, and increased reactive oxygen species (ROS) production in the liver, drive MASH pathogenesis [2]. It is likely, therefore, that any effective treatment will require a multifaceted approach; however, many treatments have focused on a single therapeutic target. Clinical trials of various drugs such as Toll-like receptor 4 (TLR4) inhibitors [5], dual peroxisome proliferator activated receptor (PPAR)-α/PPAR-γ agonists [6], farnesoid X receptor agonists [7,8], acetyl CoA carboxylase inhibitors [9], and CCR2/5 inhibitors [10,11] are currently underway or completed. Data thus far suggest that preventing liver fibrosis improves the prognosis of MASH, highlighting the importance of anti-fibrotic medications in clinical care [[12], [13], [14]]. Defective liver regeneration also contributes to the progression of chronic liver diseases [15,16]. Indeed, a fatty liver has been associated with stagnant liver regeneration [[17], [18], [19], [20]], but its molecular mechanism has not been fully understood, and there is no established therapeutic strategy. Novel therapeutics with both antifibrotic activity and the capacity to support liver regeneration, could have great potential for patients with MASH.

Heme oxygenase-1 (HO-1) is a protein that responds to oxidative stress; it is a pleiotropic regulator of inflammatory signaling programs by generating carbon monoxide (CO) and bilirubin, both of which are biologically active end products [[21], [22], [23], [24]]. Mice overexpressing HO-1 exhibit suppressed MASH pathogenesis [25,26], thus supporting a potential association between MASH and the HO-1/CO system, and suggesting that CO might function as an endogenous factor regulating MASH onset and development. Indeed, CO has potent organ protective effects via its anti-oxidative [27,28], anti-inflammatory [29], and anti-apoptotic actions [30,31]. The mechanisms of these functions of CO are diverse, such as inhibition of damage-associated molecular patterns (DAMPs)/TLR4 pathway [[32], [33], [34]] and induction of PPAR-γ and AMP-activated protein kinase (AMPK) [35,36]. Interestingly, many of these functions align with the therapeutic targets for MASH. Thus, we posit that exogenous CO could simultaneously regulate the various factors that contribute to MASH development and restain its pathological advancement.

We previously developed a CO-enriched cell therapy in which CO bound to most of hemoglobin in red blood cells (RBCs), called CO-bound RBCs (CO-RBCs) [36,37]. CO-RBCs maintain the multifaceted biological activities of CO, and thereby exert excellent organ protection against liver ischemic/reperfusion injury following resuscitation from massive hemorrhagic shock [34] and acute kidney injury [36]. In addition, CO-RBCs not only supply CO to tissues, but also function as oxygen carriers after CO release; therefore, it is unlikely that they reduce oxygenated hemoglobin levels in the blood [38]. We thus conclude that hemoglobin-based CO delivery systems exhibit the persistent, multifaceted biological activities of CO *in vivo*, and thus have the potential as therapeutic agents for MASH. In this study, we aimed to determine the effect of CO-RBCs on MASH pathogenesis and its mechanisms. To do so, we used two mouse models in which we induced MASH with a methionine-choline deficient diet (MCDD) or a high-fat diet (HFD).

## 2 Results

### 2.1 The HO-1/CO system is activated in MASH livers

**Fig. 1:**
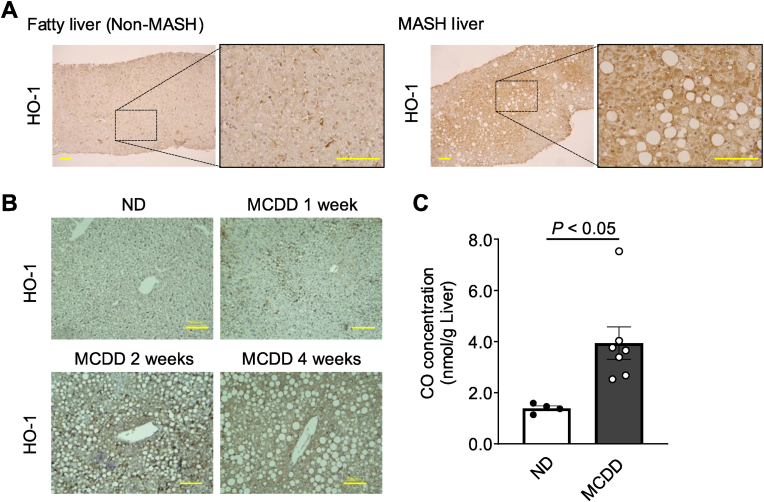
**Activation of the HO-1/CO system in the MASH liver.** (A) Representative images of immunostaining for HO-1 in human liver tissue sections made from biopsies of patients with MASH (right panel) and patients with a fatty liver (Non-MASH) (left panel). Scale bars, 100 μm; the black box represents the field of interest. (B) Representative images of immunostaining for HO-1 in murine liver tissue sections taken from normal mice fed a normal diet (ND) and mice with MASH induced with an MCDD for 1, 2 and 4 weeks (scale bars, 100 μm). (C) Carbon monoxide (CO) concentration in the livers of ND-fed mice (n = 4) and 2-week MCDD-fed mice (n = 7). Results are expressed as the mean ± S.E.

We first wanted to investigate whether the HO-1/CO system is activated in the liver of humans and mice with MASH. To do so, we performed an immunohistochemical staining for HO-1 in liver sections generated from human liver biopsy samples and MCDD-induced MASH model mice [39]. We saw significant increases in liver HO-1 expression in both the human and murine liver samples compared to fatty liver patients without MASH and healthy mice, respectively (Fig. 1A and B). The increased expression of HO-1 in MASH model mice resulted in a 2-fold increase in CO levels in the liver compared to those in healthy mice (Fig. 1C). These data suggest that liver HO-1 levels increase as MASH progresses, resulting in increased CO production in the liver.

### 2.2 CO-RBCs exhibit hepatoprotective activity in MCDD-induced MASH mice

**Fig. 2:**
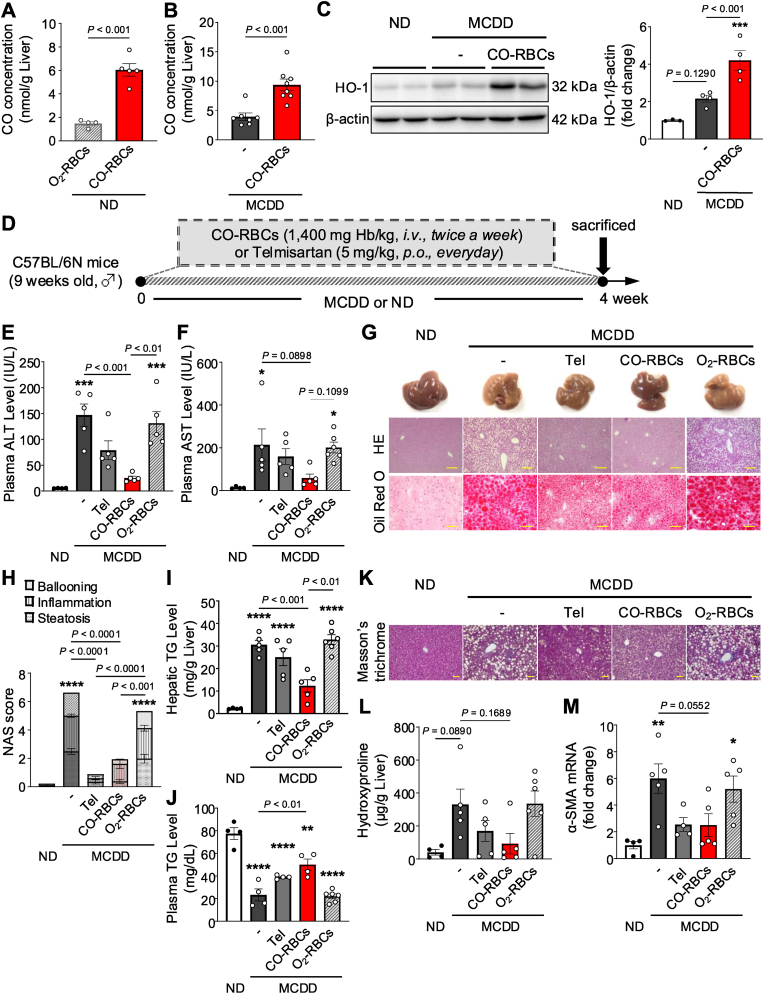
**Suppressive effect of CO-RBCs on MCDD-induced MASH.** CO concentrations in the livers of (A) ND-fed mice and (B) MCDD-fed mice, 90 min after intravenous administration of CO-RBCs, O_2_-RBCs, or saline (−) (n = 4–8/group). MCDD-fed mice were evaluated 2 weeks after the start of MCDD feeding. (C) Western blot analysis (left panel) and quantitation (right panel) of HO-1 expression in the livers of ND-fed and MCDD-fed mice exposed or not twice weekly for 2 weeks to CO-RBCs, 2 weeks after MASH induction (n = 3–4/group). (D) Schematic of the experimental protocol to determine the effect of CO-RBCs or Telmisartan (Tel) in MCDD-fed mice. Plasma levels of (E) ALT and (F) AST, (I) hepatic and (J) plasma TG levels, (L) hepatic hydroxyproline content, and (M) hepatic α-SMA mRNA levels were measured 4 weeks after the start of CO-RBCs, O_2_-RBCs, Tel or saline control (−) treatment (n = 4–6/group). (G) Representative liver tissues from ND-fed mice and MCDD-fed mice treated with saline (−), Tel, CO-RBCs, or O_2_-RBCs (top panel) and photomicrographs of hematoxylin and eosin (HE) staining (middle panel; scale bars, 100 μm) and oil Red O staining (lower panel; scale bars, 100 μm) of liver sections. (H) The NAS score was quantified with HE staining (n = 4–5/group). (K) Representative Masson's trichrome stained liver sections obtained from the same groups of mice shown in (G) (scale bars, 100 μm). Results are expressed as the means ± S.E. *P < 0.05, **P < 0.01, ***P < 0.001, ****P < 0.0001 vs. ND-fed group.

Determining that CO is produced in the liver in MASH pathology to ameliorate MASH, we next used CO-RBCs as a CO donor in the liver, and evaluated their therapeutic effect in MCDD-induced MASH model mice. First, to assess the efficiency of CO-RBCs in delivering CO to the liver, we administered CO-RBCs to healthy and MCDD-induced MASH model mice at a dose of 1400 mg Hb/kg, and then monitored liver CO levels 90 min later. CO-RBCs delivery caused a 3-fold and 2-fold increase in hepatic CO levels in healthy mice and MASH model mice, respectively (Fig. 2A and B). Interestingly, we also saw a 2-fold increase in HO-1 expression in the livers of mice treated with CO-RBCs (Fig. 2C), suggesting that the CO-RBCs were not only delivered CO to the liver but also increased endogenous liver CO production.

Next, we investigated whether CO-RBCs could prevent the development of MASH that we induced in mice by MCDD feeding (Fig. 2D). We used Telmisartan (Tel) as a positive control due to its known ability to function as a partial PPAR-γ agonist and to prevent the development of MASH by inhibiting hepatic lipid accumulation [40,41]. Liver weights decreased in all MCDD-fed mice compared to mice fed a normal diet (ND), with no significant differences observed in dietary intake between the two groups (Table S1). MCDD feeding for 4 weeks significantly increased the plasma levels of alanine aminotransferase (ALT) and aspartate aminotransferase (AST) compared to the ND-fed mice (Fig. 2E and F). Hematoxylin and eosin (HE) staining of liver sections of the MCDD-fed group revealed pathological characteristics of MASH, such as ballooning of hepatocytes and infiltration of inflammatory cells, while oil red O staining revealed the presence of intrahepatic lipids (Fig. 2G and H). All of these markers were significantly reduced when CO-RBCs or Tel were administered compared to mice not receiving these, but not with O_2_-RBCs (which served as a negative control) (Fig. 2E–J).

To determine whether CO-RBCs also influenced the extent of liver fibrosis, we performed Masson's trichrome staining of liver sections and measured the liver hydroxyproline content (Fig. 2K and L). An increase in the staining area and hydroxyproline content was observed in MCDD-fed mice, and tended to be inhibited by CO-RBCs or Tel. Furthermore, quantitative RT-PCR analysis showed that the mRNA levels of α-smooth muscle actin (SMA), a marker of fibrosis, increased with MCDD feeding and were significantly suppressed by CO-RBCs or Tel treatment (Fig. 2M). These data suggest that CO derived from CO-RBCs effectively suppresses the progression of MCDD-induced MASH pathology to an extent comparable to daily administration of Tel.

### 2.3 Involvement of Kupffer cells in the inhibition of MASH progression by CO-RBCs

**Fig. 3:**
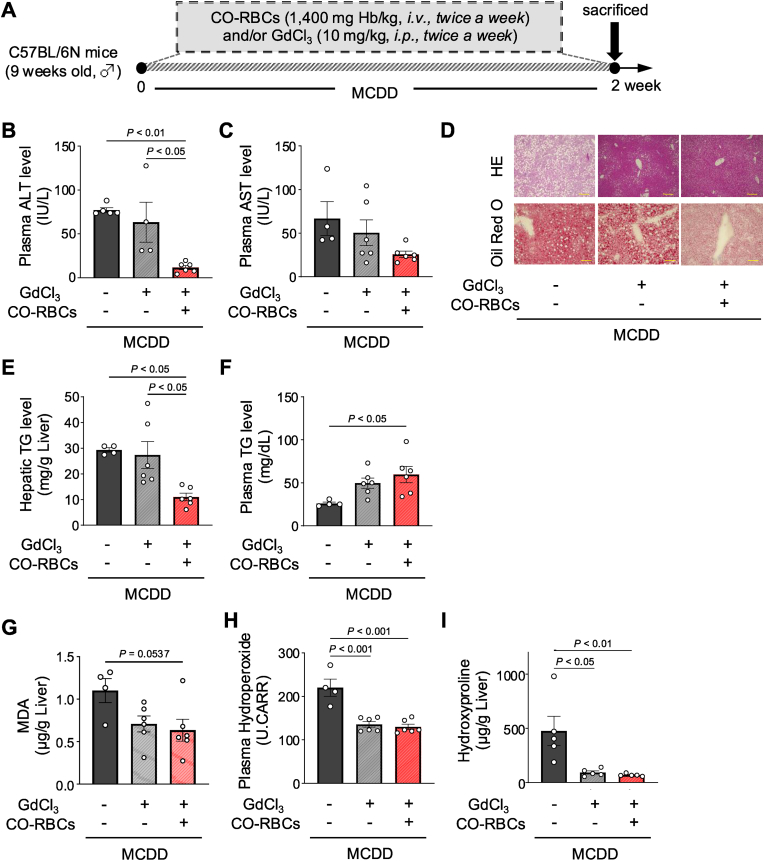
**Involvement of Kupffer cells in the suppressive effect of CO-RBCs on the progression of MASH.** (A) Schematic of the experimental protocol to evaluate the involvement of Kupffer cells in the suppressive effect of CO-RBCs on MASH progression. Plasma (B) ALT and (C) AST levels, (E) liver and (F) plasma TG levels, (G) hepatic malondialdehyde (MDA) content, (H) plasma hydroperoxide content, and (I) hepatic hydroxyproline content were determined in MCDD-fed MASH mice exposed or not to GdCl_3_ and CO-RBCs (n = 4–6/group). (D) Representative photomicrographs of hematoxylin and eosin (HE) staining (top panel; scale bars, 100 μm) and oil Red O staining (bottom panel; scale bars, 50 μm) of liver sections obtained from the livers of the groups of mice shown in B–I. Results are expressed as the means ± S.E.

Activated Kupffer cells are key markers of an inflammatory response and oxidative stress during MASH development [42,43]. We therefore aimed to determine the involvement of Kupffer cells in the restorative effect of CO-RBCs on MASH pathophysiology. First, we eliminated Kupffer cells in MCDD-induced MASH mice using gadolinium chloride (GdCl_3_), a known Kupffer cell inhibitor [34,44] (Fig. 3A). GdCl_3_ had no impact on the elevated plasma ALT and AST levels, lipid accumulation, and increased liver TG levels (Fig. 3B–F) but significantly reduced the accumulation of the oxidative stress markers such as malondialdehyde (MDA) and hydroperoxide, and the fibrosis marker hydroxyproline (Fig. 3G–I). We thus suppose that Kupffer cells have a minor role in liver fat accumulation and hepatocyte injury in this MASH model but are rather involved in mediating oxidative stress and fibrosis. Interestingly, the administration of CO-RBCs to GdCl_3_-treated MCDD-fed MASH mice improved the plasma levels of ALT and AST and hepatic TG (Fig. 3B–F), suggesting that these effects of CO-RBCs were independent of Kupffer cells.

### 2.4 The therapeutic action of CO-RBCs on MCDD-induced MASH

**Fig. 4:**
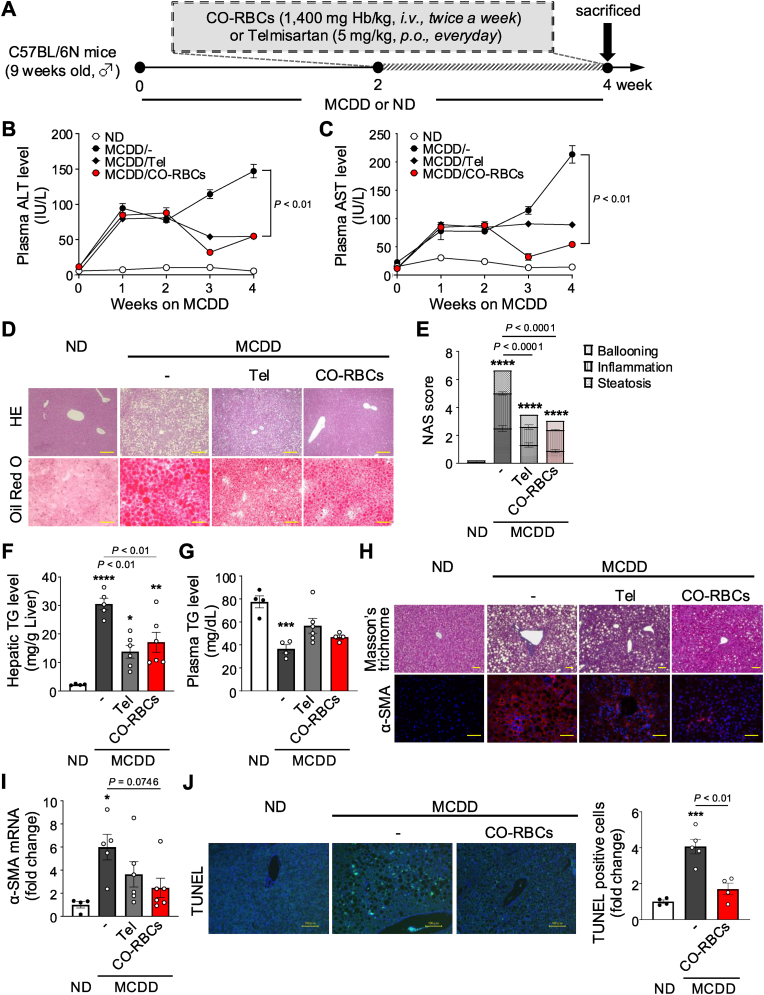
**Therapeutic effect of CO-RBCs on MCDD-induced MASH.** (A) Schematic of the experimental protocol to determine the effect of CO-RBCs, Telmisartan (Tel) or saline (−) in ND-fed mice and MCDD-fed mice. Plasma (B) ALT and (C) AST levels were monitored for 4 weeks (n = 4–6/group). (D) Representative photomicrographs of hematoxylin and eosin (HE) staining (upper panel; scale bars, 100 μm) and oil Red O staining (lower panel; scale bars, 50 μm) of liver sections. (E) The NAS score was quantified with HE staining (n = 4–5/group). (F) Hepatic and (G) plasma TG levels and (I) hepatic α-SMA mRNA levels were measured 2 weeks after the start of CO-RBCs, Tel, or saline (−) treatment (n = 4–6/group). (H) Representative photomicrographs of Masson's trichrome staining (top panel; scale bars, 100 μm) and α-SMA immunostaining (bottom panel; scale bars, 25 μm) of sections taken from the livers of mice from each experimental group. (J) Representative photomicrographs of TUNEL staining of sections taken from the livers of mice from each experimental group (scale bars, 100 μm) (left panel), and the respective quantification (right panel) where TUNEL-positive cells were defined as double-positive cells for TUNEL and DAPI (n = 4–5/group). Results are expressed as the means ± S.E. *P < 0.05, **P < 0.01, ***P < 0.001, ****P < 0.0001 vs. ND-fed group.

We next monitored hepatic TGs, lipid accumulation, and hydroxyproline levels during MCDD feeding to establish the optimal starting point for CO-RBCs-mediated therapeutic intervention. These parameters increased as the MCDD feeding period progressed, and reached a plateau after 2 weeks (Figs. S1A–D). We thus concluded that therapeutic intervention with CO-RBCs or Tel should be initiated at 2 weeks (Fig. 4A). During the intervention period, the plasma ALT level gradually declined in response to Tel treatment, while the plasma AST level remained constant for up to 4 weeks (Fig. 4B and C). By contrast, the administration of CO-RBCs from 2 weeks of MCDD feeding significantly suppressed these elevations of plasma ALT and AST levels after 4 weeks (Fig. 4B and C). Additionally, the plasma levels of ALT and AST and hepatic TG were reduced by CO-RBCs in a dose-dependent manner (Figs. S2A–D). CO-RBCs and Tel markedly improved visual signs of histological liver damage and reduced liver TG accumulation in MASH mice (Fig. 4D–G). Interestingly, CO-RBCs showed a greater ability to inhibit liver fibrosis than Tel (Fig. 4H and I). Furthermore, TUNEL staining showed that the CO-RBCs markedly inhibited MCDD-induced liver apoptosis (Fig. 4J). These results suggest that CO-RBCs are an effective treatment for MASH.

### 2.5 CO-RBCs have anti-inflammatory and antioxidant effects in MCDD-fed MASH mice

**Fig. 5:**
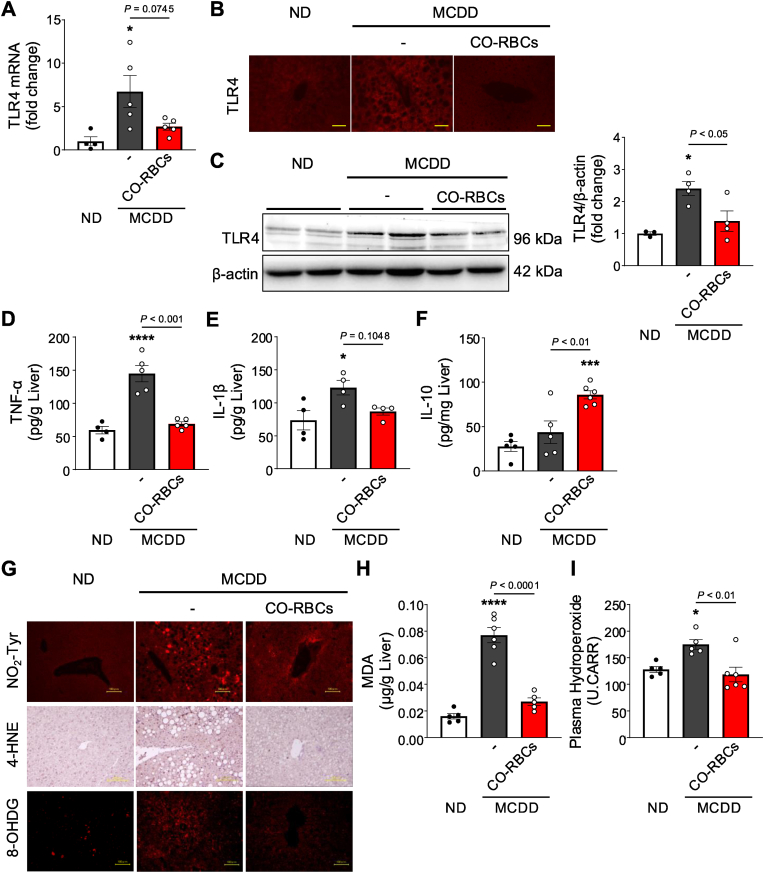
**Effects of anti-inflammatory and anti-oxidative stress of CO-RBCs on MCDD-induced MASH.** TLR4 expression in the liver was evaluated by (A) qRT-PCR, (B) immunofluorescence (scale bars, 100 μm) and (C) western blotting in ND-fed mice and MCDD-fed mice trated with saline (−) or CO-RBCs (n = 3–5/group). (D) TNF-α, (E) IL-1β and (F) IL-10 protein levels in the liver were quantified by ELISA (n = 4–6/group). (G) Representative photomicrographs of nitrotyrosine staining (NO_2_-Tyr; upper panel; scale bars, 100 μm), 4-hydroxynonenal staining (4-HNE; middle panel; scale bars, 100 μm) and 8-hydroxydeoxyguanosine staining (8-OHDG; lower panel; scale bars, 100 μm) of liver sections taken from the livers of mice in each experimental group. (H) Hepatic malondialdehyde (MDA) content and (I) plasma hydroperoxide content in the liver of mice from each experimental group (n = 5–6/group). Mice used in the experiment received the same treatment as in Fig. 4. Results are expressed as the mean ± S.E. *P < 0.05, ***P < 0.001, ****P < 0.0001 vs. ND-fed group.

Chronic TLR4-mediated inflammation and ROS production in the liver markedly contribute to MASH pathogenesis [45,46]. As such, we investigated the effects of CO-RBCs on liver TLR4 expression in MCDD-fed mice. MCDD feeding markedly increased liver TLR4 expression, which was significantly reduced by CO-RBCs (Fig. 5A–C). Furthermore, elevations in TNF-α and IL-1β levels (both inflammatory cytokines) in the liver induced by MCDD feeding were suppressed by the CO-RBCs (Fig. 5D and E). By contrast, IL-10, an anti-inflammatory cytokine, was significantly increased in mice treated with CO-RBCs (Fig. 5F).

We further investigated the effect of CO-RBCs on oxidative stress in MCDD-fed MASH mice. Liver sections from these mice showed marked accumulations of nitrotyrosine (NO_2_-Tyr), 4-hydroxynonenal (4-HNE), and 8-hydroxy-2′-deoxyguanosine (8-OHdG), which are markers of oxidative stress, compared to ND-fed mice (Fig. 5G). CO-RBCs administration resulted in a decrease in the levels of these markers (Fig. 5G) as well as the levels of liver MDA and plasma hydroperoxides (markers of lipid oxidation) (Fig. 5H and I). We therefore conclude that CO-RBCs exert their anti-inflammatory and antioxidant effects on MCDD-induced liver injury by inducing changes in macrophage properties.

### 2.6 CO-RBCs restore fatty acid oxidation in MCDD-fed MASH mice

**Fig. 6:**
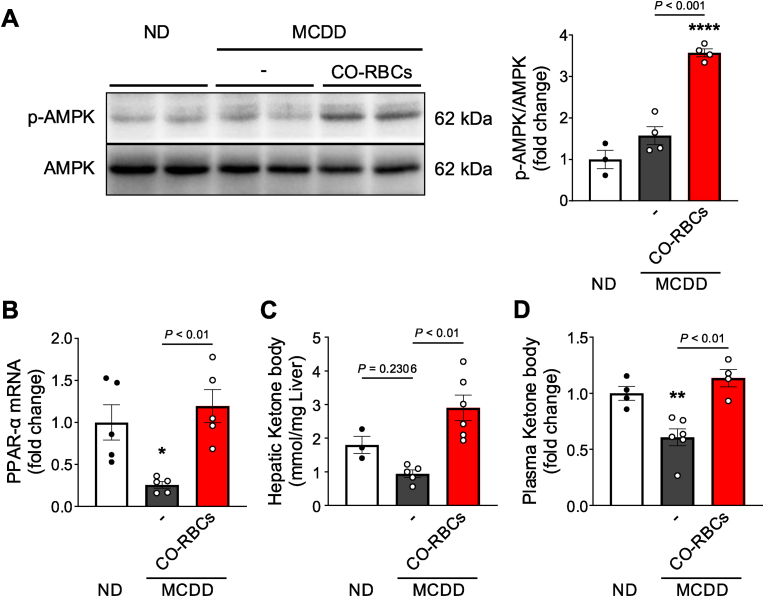
**Enhancement of fatty acid oxidation (FAO) effects of CO-RBCs on MCDD-induced MASH.** (A) Hepatic AMPK activity in ND-fed mice and MCDD-fed mice exposed to saline (−) or CO-RBCs, was determined by western blotting (n = 3–4/group). (B) Hepatic PPAR-α mRNA levels, as determined by qRT-PCR (n = 5/group) and (C) hepatic and (D) plasma ketone body content (n = 3–6/group) was measured in the livers of mice from all experimental groups 2 weeks after the start of CO-RBCs or saline (−) treatment. Mice used in the experiment received the same treatment as in Fig. 4. Results are expressed as the means ± S.E. *P < 0.05, **P < 0.01, ****P < 0.0001 vs. the ND-fed group.

In our next analyses, we focused on the effects of CO-RBCs on the AMPK pathway as phosphorylated AMPK leads to the activation of fatty acid oxidation (FAO) [47,48]. By western blotting, we saw that the liver *p*-AMPK/AMPK ratio was significantly increased following CO-RBCs administration in MCDD-fed mice, suggesting the activation of liver AMPK by CO-RBCs (Fig. 6A). Furthermore, the liver levels of PPAR-α, an FAO-related gene that regulates molecules downstream of AMPK [49,50], and ketone bodies that are end products of FAO, were significantly reduced in the MCDD-fed mice. The levels of these molecules were restored upon CO-RBCs treatment (Fig. 6B–D). These findings imply that CO-RBCs promote FAO by enhancing AMPK phosphorylation and help to ameliorate hepatic lipid accumulation in MCDD-induced MASH mice.

### 2.7 CO-RBCs have therapeutic effects in HFD-induced MASH mice

**Fig. 7:**
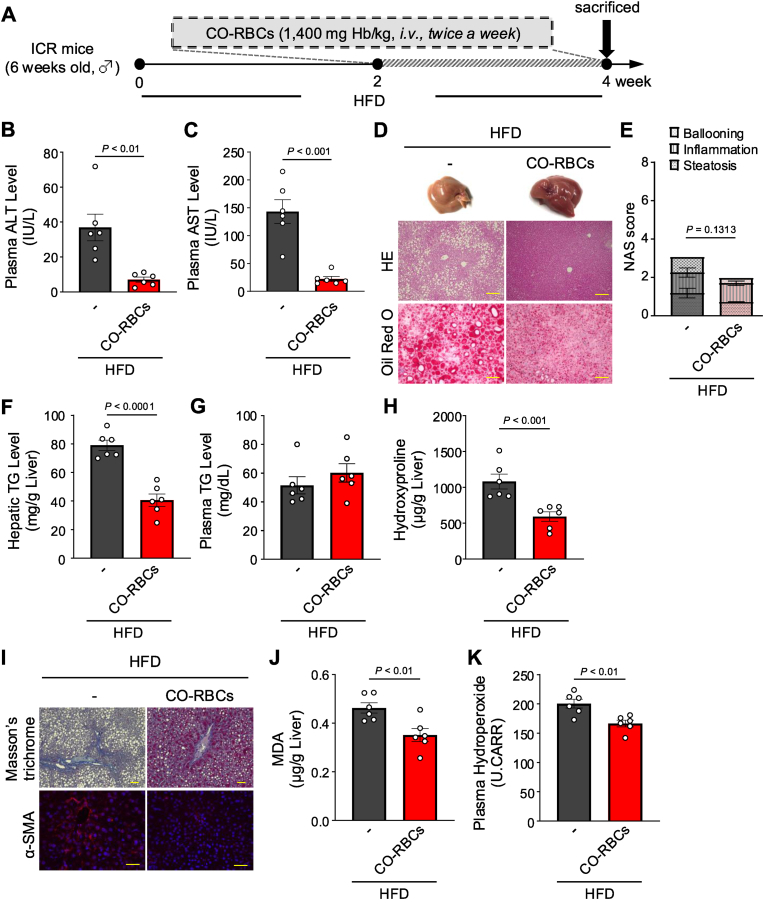
**Therapeutic effects of CO-RBCs on a high-fat diet (HFD)-induced MASH.** (A) Schematic of the experimental protocol to determine the effect of CO-RBCs in HFD-induced MASH model mice. Plasma (B) ALT and (C) AST levels, (F) liver and (G) plasma TG levels, (H) hepatic hydroxyproline content, (J) hepatic malondialdehyde (MDA) content, and (K) plasma hydroperoxide content were measured 2 weeks after the start of CO-RBCs or saline (−) treatment (n = 6/group). (D) Representative liver tissues (top panel), photomicrographs of hematoxylin and eosin (HE) staining (middle panel; scale bars, 100 μm) and oil Red O staining (bottom panel; scale bars, 100 μm) of sections taken from the liver of mice in all experimental groups. (E) The NAS score was quantified with HE staining (n = 6/group). (I) Representative photomicrographs of Masson's trichrome (top panel; scale bars, 100 μm) and α-SMA immunostaining (bottom panel; scale bars, 25 μm) of liver sections taken from mice of all experimental groups, 2 weeks after the start of CO-RBCs administration. Results are expressed as the means ± S.E.

In our following assays, we focused on the hepatoprotective effects of CO-RBCs in HFD-induced MASH mice (Fig. 7A). There were no significant differences between the CO-RBCs treated and non-treated HFD mice in terms of their body weight, liver weight, food intake, and epididymal fat weight (Table S2). However, CO-RBCs administration significantly suppressed the increase in plasma ALT and AST levels induced by HFD feeding (Fig. 7B and C), and reduced the extent of HFD-induced liver damage, liver lipid accumulation (Fig. 7D and E), and liver TG accumulation (Fig. 7F). Meanwhile, we saw no significant differences in plasma TG concentrations between the groups (Fig. 7G). Adipocyte hypertrophy in MASH pathology has been reported to contribute to inflammation and lipid accumulation, and CO-RBCs significantly ameliorated HFD-induced adipocyte hypertrophy (Fig. S3). Furthermore, CO-RBCs significantly suppressed the increase in hepatic hydroxyproline content, which is indicative of fibrosis (Fig. 7H), which we confirmed by Masson's trichrome staining and α-SMA immunofluorescence staining of liver sections (Fig. 7I). Finally, similar to our findings made in MCDD-fed MASH mice, CO-RBCs treatment also significantly suppressed the accumulation of liver MDA and plasma hydroperoxides (Fig. 7J and K), which are indicators of oxidative stress. These results support that CO-RBCs also have a potent hepatoprotective effect in HFD-induced MASH model mice.

### 2.8 CO-RBCs induce Akt-independent liver regeneration in HFD-fed MASH mice

**Fig. 8:**
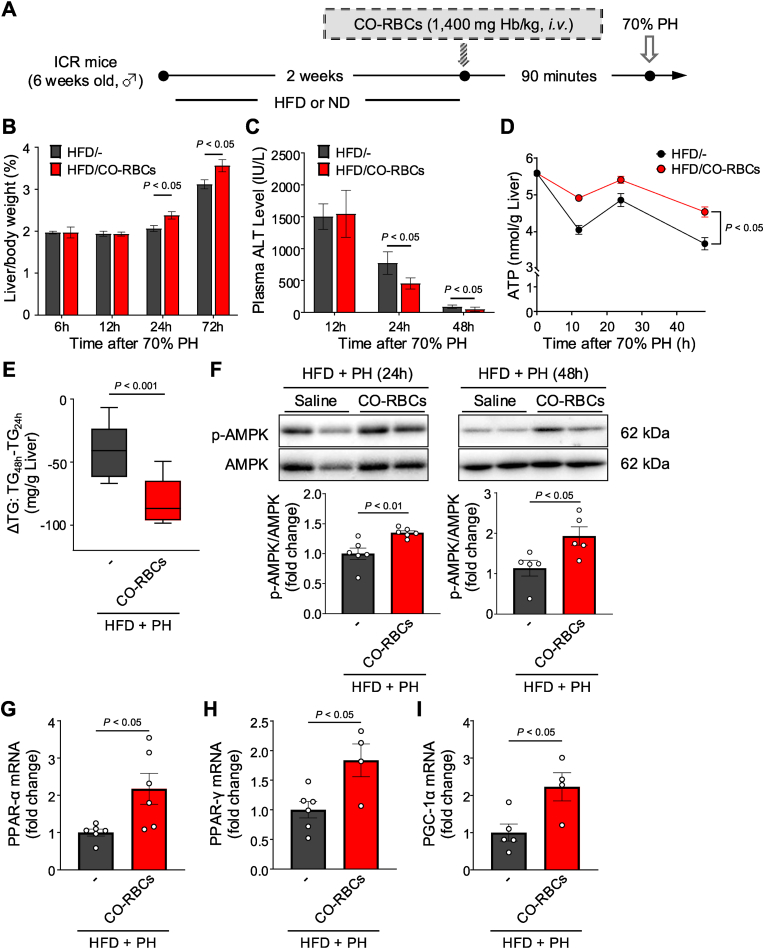
**Therapeutic effect of CO-RBCs on a high-fat diet (HFD)-induced liver regenerative failure.** (A) Schematic of the experimental protocol to determine the hepatic regenerative effect of CO-RBCs in normal diet (ND)-fed mice or high fat diet (HFD)-fed mice. (B) Liver weight as a percentage of body weight at 6, 12, 24 and 72 h after 70 % partial hepatectomy (PH) in HFD-fed mice treated with CO-RBCs or saline (−) (n = 4–5/group). (C) Plasma ALT levels at 12, 24 and 48 h after PH in mice from all experimental groups (n = 4/group). (D) Adenosine triphosphate (ATP) levels were monitored in the livers of mice from all experimental groups, for 48 h after PH (n = 3–5/group). The liver (E) TG levels and (F) AMPK activities were evaluated 24 and 48 h after PH in mice from all experimental groups, and the ΔTG was calculated (n = 5–9/group). Liver (G) PPAR-α, (H) PPAR-γ, and (I) PGC-1α mRNA levels were measured by qRT-PCR 24 h after PH in mice from all experimental groups (n = 4–6/group). Results are expressed as the means ± S.E.

The reduced regenerative capacity of the fatty liver exacerbates MASH pathology in affected patients [17,18,20]. We thus investigated the effects of CO-RBCs on liver regeneration after 70 % partial hepatectomy (PH) in HFD-induced MASH mice (Fig. 8A). We saw enhanced liver regeneration in HFD-fed and ND-fed mice treated with CO-RBCs (Fig. 8B and Fig. S4A). Furthermore, plasma ALT levels in HFD-fed mice were significantly reduced 24 h after CO-RBCs treatment, coinciding with improved liver regeneration (Fig. 8C). We also detected increased expression of the cell cycle progression markers CyclinD1 and Ki67 [51,52] in ND-fed mice treated with CO-RBCs (Figs. S4B and C). This effect was accompanied by the activation of Akt, a known inducer of liver regeneration [53] (Fig. S4D). Despite the enhanced liver regeneration in CO-RBCs-treated HFD-fed MASH mice, we saw minimal activation of Akt in this group (Fig. S4E), suggesting that CO-RBCs induce liver regeneration via a different mechanism.

Indeed, after measuring ATP and TG levels in the liver of HFD-fed MASH mice, we saw enhanced ATP production and TG degradation in CO-RBCs treated versus untreated mice (Fig. 8D and E). Furthermore, CO-RBCs activated AMPK and increased the expressions of PPAR-α, PPAR-γ, and PGC-1α, which are downstream factors of AMPK (Fig. 8F–I). These data suggest that CO-RBCs promote liver regeneration in HFD mice using stored lipids as an energy source.

### 2.9 CO-RBCs induce an anti-inflammatory effect in RAW264.7 cells and improve lipid metabolism in HepG2 cells

**Fig. 9:**
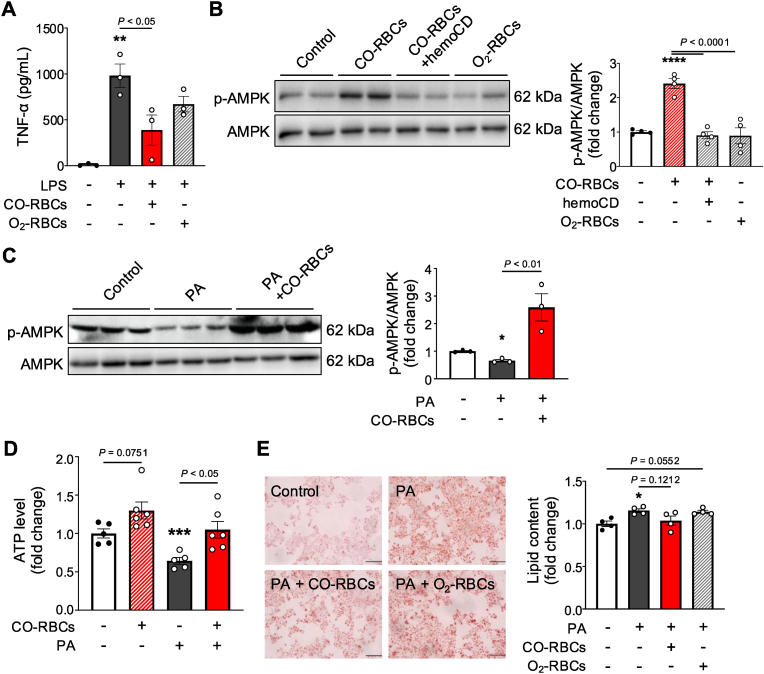
**Anti-inflammatory effect on RAW264.7 cells and improvement of lipid metabolism in HepG2 cells by CO-RBCs.** (A) TNF-α levels in the culture supernatant of RAW264.7 cells 2 h after LPS treatment and CO-RBCs or O_2_-RBCs exposure, or no treatment (−) (n = 3/group). CO-RBCs (0.5 mg Hb/ml) or O_2_-RBCs (0.5 mg Hb/mL) were administered 24 h before LPS treatment. (B) AMPK activity in untreated HepG2 cells was determined by western blotting 12 h after CO-RBCs (0.5 mg Hb/mL), a mixture of CO-RBCs (0.5 mg Hb/mL) and hemoCD (30 μM), or O_2_-RBCs (0.5 mg Hb/mL) exposure (n = 4/group). (C) AMPK activity in HepG2 cells was determined by western blotting 12 h after palmitic acid (PA; 200 μM) exposure (n = 3/group). CO-RBCs (0.5 mg Hb/mL) were administered 1 h after PA treatment. (D) ATP levels in HepG2 cells were evaluated 24 h after CO-RBCs exposure (0.5 mg Hb/ml) (n = 5–6/group). PA (200 μM) was administered 24 h before CO-RBCs treatment. (E) Representative photomicrographs (left panel) of oil Red O-stained HepG2 cells, 24 h after CO-RBCs (0.5 mg Hb/ml) or O_2_-RBCs (0.5 mg Hb/mL) treatment (scale bars, 100 μm). PA (100 μM) was administered simultaneously with CO-RBCs or O_2_-RBCs. Intracellular lipid content was quantified (right panel) by measuring the absorbance at 490 nm derived from oil Red O staining (n = 4/group). *P < 0.05, **P < 0.01, ***P < 0.001, ****P < 0.0001 vs. the non-treatment group. Results are expressed as the means ± S.E.

In our final set of analyses, we wanted to verify the anti-inflammatory effects of CO-RBCs *in vitro*. We first measured TNF-α production after lipopolysaccharide (LPS) stimulation in the RAW264.7 macrophage cell line [54], and in the absence and presence of CO-RBCs. Pretreatment with CO-RBCs prevented the increase in TNF-α induced by LPS, while O_2_-RBCs did not (Fig. 9A).

We also examined the effect of CO-RBCs on lipid metabolism by evaluating changes in AMPK activity in HepG2 cells. We used O_2_-RBCs and co-treatment of CO-RBCs and hemoCD, a CO scavenger [55], as controls. We saw AMPK activation after CO-RBCs treatment, but not in any of the other conditions, indicating the crucial role of CO in the action of CO-RBCs (Fig. 9B). To further confirm the action of CO-RBCs, we performed similar experiments using HepG2 cells treated with palmitic acid (PA), an inducer of lipotoxicity [56,57]. Here we also saw AMPK induction by CO-RBCs and a concomitant increase in intracellular ATP levels (Fig. 9C, D) and a decrease in lipid accumulation (evaluated by oil Red O staining; Fig. 9E). Taken together, CO-RBCs seem to exert anti-inflammatory effects on macrophages and ameliorate lipid accumulation in hepatocytes.

## 3 Discussion

Recent work by Hong et al. led to the development of nanoparticles containing an HO-1 inducer for MASH treatment, which decreased lipid accumulation and cytokine production [58]. These findings signaled to the community that HO-1 protects the liver in the MASH condition. Later, Bessa et al. reported that carboxyhemoglobin blood levels were increased in MASH patients, and that there was a positive correlation between HO-1 expression and plasma carboxyhemoglobin levels [59]. Inspired by these and other studies, we first checked that HO-1 was induced in both MASH patients and MCDD-induced MASH mice. Alongside, we saw that CO levels were also significantly elevated in MASH mice. From here, this study aimed to develop and validate CO-RBCs as a bioinspired CO donor with therapeutic efficacy in MCDD-induced and HFD-induced MASH mice. Through our *in vivo* and *in vitro* analyses, we identified both preventive and therapeutic effects of CO-RBCs in these MASH mouse models. Mechanistically, CO-RBCs inhibited Kupffer cell activation with anti-inflammatory and antioxidant effects, suppressed fat accumulation in hepatocytes via AMPK/PPAR-α-stimulated FAO, and promoted fatty liver regeneration via AMPK activation/lipid metabolism but not Akt.

We first investigated the potential preventive effect of CO-RBCs and O_2_-RBCs on MCDD-induced MASH model mice and found that CO-RBCs greatly reduced disease progression, but O_2_-RBCs did not (Fig. 2). We then administered CO-RBCs and O_2_-RBCs biweekly to mice at the stage where the MASH pathology had already developed (2 weeks after the start of the MCDD feeding) and found that the CO-RBCs improved the MASH pathology, including hepatocellular damage and fat accumulation in the liver (Fig. 4). Additionally, CO-RBCs significantly slowed the progression of hepatic fibrosis. The results of recent clinical trials of MASH therapies have demonstrated that hepatic fibrosis determines the prognosis of MASH [13,14]. We achieved similar therapeutic effects from CO-RBCs treatment in HFD-induced MASH mice (Fig. 7). These findings indicate that CO-RBCs not only prevent MASH progression, but also improve hepatic fibrosis. Interestingly, exogenous CO from inhalation or CO-releasing agents increased HO-1 expression in the liver in the previous reports [60,61]. Our results were also consistent, as we observed a marked increase in HO-1 expression when CO-RBCs were administered to MASH model mice. These results suggest that exogenous CO derived from CO-RBCs increases HO-1 expression, which in turn may increase endogenous CO production, resulting in sustained effects of CO.

Increased fatty acid metabolism is largely responsible for the suppression of fat accumulation [62]. Here, our CO-RBCs-treated mice showed enhanced β-oxidation, increased β-hydroxybutyrate levels, and AMPK activation (Fig. 6). We thus hypothesised that CO-RBCs stimulate β-oxidation by activating the AMPK pathway, suppressing fat accumulation in the liver as a result. Indeed, several studies have reported that AMPK activation is effective in inhibiting MASH progression [47,48,63]. The precise mechanism by which CO activates AMPK now warrants further exploration. However, previous work showed that CO reduces intracellular ATP in glial cells [64]. Given that AMPK is activated in response to reduced intracellular ATP levels [65], we presume that AMPK activation by CO might be mediated by a transient decrease in intracellular ATP levels. Furthermore, CO-RBCs increased PPAR-α mRNA expression in the MASH liver (Fig. 6B). As PPAR-α expression occurs downstream of AMPK [49], we propose that CO released from CO-RBCs might indirectly induce PPAR-α via AMPK activation under MASH conditions. Such a unique action of CO also contributed to the hepatocyte proliferation in fatty liver. As shown in Fig. 8B and Fig. S4A, CO-RBCs promoted regeneration in both healthy livers and fatty livers, but the mechanisms were different. The promotion of liver regeneration by CO-RBCs was accompanied by Akt activation for healthy livers and AMPK activation for fatty livers. Based on present limited data, it is difficult to explain the precise mechanism of this difference, but proliferation of hepatocyte in damaged liver may require more energy than healthy condition through AMPK activation. Indeed, Huang et al. reported that liver regeneration in hepatitis model mice exposed to carbon tetrachloride was mediated by AMPK activation [66]. Therefore, an activation of FAO by AMPK may play an important role for the recovery of impaired liver regeneration in MASH.

Activated Kupffer cells induce inflammatory cytokine and ROS production during MASH development [42,43], thus rendering Kupffer cells as a potential therapeutic target. To ascertain whether CO-RBCs inhibit Kupffer cell activation, we investigated the effect of CO-RBCs after Kupffer cell depletion with GdCl_3_ in MCDD-induced MASH mice (Fig. 3). Kupffer cell depletion alone had a minimal effect on plasma ALT and AST levels or liver TGs, but significantly inhibited the accumulation of markers of oxidative stress and fibrosis in MASH mice. We thus conclude that in our experimental model, Kupffer cells are predominantly involved in mediating oxidative stress and fibrosis. When we administered CO-RBCs to mice pre-treated with GdCl_3_, their plasma AST and ALT levels and hepatic TG improved, but their oxidative stress or liver fibrosis in the liver were not inhibited (Fig. 3). These results, together with our *in vitro* analyses in RAW264.7 cells and HepG2 cells (Fig. 9), indicate that CO-RBCs may act on both hepatocytes and Kupffer cells.

During MASH, damaged hepatocytes increase the secretion of food-derived PA and DAMPs that alongside TLR4 activation promotes chronic inflammation and oxidative stress in the liver [67]. Interestingly, the severity of liver dysfunction and fibrosis in HFD-fed TLR4 knockout mice was lower than in WT mice [45,46]. These findings indicate that the TLR4 pathway has a notable impact on MASH progression. Our analyses showed that TLR4 expression was upregulated in MCDD-fed MASH mice, leading to the production of inflammatory cytokines and an increase in oxidative stress markers. CO-RBCs markedly suppressed these effects (Fig. 5). We previously reported that CO-RBCs effectively decrease liver ischemic/reperfusion injury caused by the resuscitation of RBCs following massive hemorrhagic shock. We found that CO inhibits TLR4 signalling in Kupffer cells and exerts anti-inflammatory effects [34]. Moreover, results from other studies showed that CO-enriched cell therapy based on hemoglobin, called CO-bound hemoglobin vesicles (CO-HbVs) have anti-inflammatory effects in animal models of inflammatory bowel disease and acute pancreatitis: CO-HbVs were able to decrease pro-inflammatory cytokines and promote the production of anti-inflammatory cytokines [68,69]. In the present study, CO-RBCs produced similar results in our MASH model mice (Fig. 5). These unique anti-inflammatory effects of CO might contribute to the therapeutic effects of CO-RBCs. By switching the phenotype of the causative immune cells, such as macrophages and T cells, from inflammatory to anti-inflammatory, it is possible to improve MASH prognosis [70,71]. More studies to elucidate the immunomodulatory effects of CO-RBCs are, therefore, warranted.

Therapeutic agents for MASH are currently under study in phase II clinical trials, including TLR4 inhibitors [5], PPAR-α and PPAR-γ dual agonists [6] and acetyl CoA carboxylase inhibitors [9]. Given that CO-RBCs not only increase AMPK activity and PPAR-α expression, but also suppress TLR4 expression and enhance IL-10 production, we believe that CO-RBCs can elicit the effects of all these experimental drugs and constitute an all-encompassing MASH treatment. Indeed, AMPK activation should produce effects comparable with acetyl CoA carboxylase inhibitors, as it inhibits the induction of acetyl CoA carboxylase and decreases fatty acid production. Recently, a partial agonist of the thyroid hormone receptor (THR) has been approved for the treatment of MASH [12,72]. This drug exerts its effect by interacting with THR and activating the transcription of proteins involved in metabolism and immunity. The combination of CO-RBCs, which activates AMPK, and this drug may have an additive effect.

It is important to note that our MASH model mice did not exhibit significantly impaired glucose tolerance. Recent reports have shown that metabolic dysfunction-associated steatotic liver disease (MASLD) and MASH can be associated with metabolic syndrome, and that affected patients often have impaired glucose tolerance [[73], [74], [75]]. Considering the clinical application of CO-RBCs, it is desirable that CO-RBCs has a therapeutic effect on MASH patients with insulin resistance and diabetes. As it has been reported that CO improves insulin resistance and the pathophysiology of animal models of diabetes [76], we expect that CO-RBCs will be effective for these MASH patients as well. Further *in vitro* and *in vivo* studies are needed in this regard.

## 4 Conclusion

CO-RBCs effectively suppressed key pathological characteristics of MASH, including liver fat accumulation, hepatocyte damage, hepatic fibrosis, and impaired liver regeneration, in two MASH mouse models. These effects were achieved through AMPK activation, PPAR-α induction, TLR4 inhibition, and IL-10 induction. These mechanisms are considered crucial for improving MASH prognosis in affected patients, thus implying that CO-RBCs might constitute a promising treatment option.

## 5 Materials and methods

### 5.1 Chemicals and materials

MCDD was purchased from MP Biomedicals (Solon, OH). HFD, namely STHD-01, was provided by EA Pharma Co., Ltd. (Tokyo, Japan). CO gas was purchased from Kumamoto Sanso Co. (Kumamoto, Japan). Tel was purchased from Tokyo Chemical Industry Co., Ltd. (Tokyo, Japan). GdCl_3_ was purchased from Sigma-Aldrich (St Louis, MO). All other chemicals were of the highest commercially available quality, and all solutions were prepared using ion-exchange water.

### 5.2 Human liver biopsy samples

Human liver biopsy samples were provided by the Department of Gastroenterology and Hepatology, Graduate School of Medical Sciences, Kumamoto University (Kumamoto, Japan), and the patient information are presented in our previous report [39]. Each patient was diagnosed with NASH but redefined as MASH according to current criteria [77]. Written informed consent was obtained from each participant. The study protocol conformed to the guidelines of the Declaration of Helsinki and was approved by the Ethics Review Committee of Kumamoto University.

### 5.3 Animal experiments

C57BL/6 N mice (male, Japan SLC Inc., Shizuoka, Japan) and ICR mice (male, Japan SLC Inc.) were reared in a temperature-controlled room with a 12-h dark/light cycle with food and water available *ad libitum*. All animal experiments were conducted in accordance with the procedures approved by the Experimental Animal Ethics Committee of Kumamoto University. After 1 week of rearing, the mice were fed MCDD or HFD for 4 weeks. CO-RBCs (1400 mg Hb/kg) and O_2_-RBCs (1400 mg Hb/kg) were administered via the tail vein. Mice were fed a CE-2 diet (CLEA Japan, Inc., Tokyo, Japan) outside the MCDD or HFD feeding period.

To evaluate liver regeneration, a mouse model with 70 % PH was created according to previous reports [78,79]. In brief, after cutting the liver cirrus of ICR mice (6 weeks old), the parts of the middle and left lobe of the liver were pushed out of the abdominal cavity by finger pressure and both lobes were debrided. Then the gallbladder was ligated using sutures and all tissue below the ligature was removed.

### 5.4 Immunohistochemical and histological analyses

Human liver biopsy and mouse liver samples were fixed with 4 % paraformaldehyde (PFA). Paraffin permeabilization treatment and paraffin embedding followed. The paraffin sections were then cut into 4 μm-thick slices using a microtome and adhered to glass slides. Then, the paraffin was removed using xylene and ethanol and incubated in Histo VT One (30 min, 95 °C; Nacalai Tesque, Kyoto, Japan) for antigen activation. Subsequently, the samples were incubated in 0.3 % hydrogen peroxide/methanol solution for 30 min, followed by blocking treatment with 4 % Block Ace (DS Pharma Biomedical, Osaka, Japan) for 15 min before primary antibody incubation (4 °C, overnight). Primary antibody labelling was visualised by the universal immuno-enzyme polymer method using a Histofine Immunohistochemistry Kit (Nichirei, Tokyo, Japan) and 3,3′-diaminobenzidine (Dojin Chemical, Kumamoto, Japan), according to the manufacturer's protocol. The slides were then observed under a microscope (BZ-8000; Keyence, Osaka, Japan). All antibodies used in this study are detailed in Table S3.

The sections were subjected to HE and Masson's trichrome staining for morphologic analysis and the detection of collagen fibres, respectively. Images of the HE staining were used for NAS score quantification and were blindly evaluated according to the Kleiner et al. method [80]. TUNEL staining to detect apoptotic cells was performed using an In Situ Cell Death Detection Kit (Roche, Mannheim, Germany). Freshly frozen tissue blocks were cut into 10-μm-thick sections and stained with oil Red O to detect lipid droplets. The staining solution was prepared by dissolving 0.3 g of oil Red O powder (Wako Pure Chemical, Ltd., Wako, Osaka, Japan) in 100 mL isopropanol, followed by a 3:2 dilution with distilled water.

### 5.5 Preparation of CO-RBCs and O_2_-RBCs

CO-RBCs and O_2_-RBCs were purified as previously described [37]. Briefly, blood samples of C57BL/6 N mice (male, 9 weeks old, Japan SLC Inc.) or ICR mice (male, 8 weeks old, Japan SLC Inc.) were centrifuged (190×*g*, 10 min, 4 °C). The RBCs suspension was washed three times with saline to remove plasma components. The Hb concentration in the washed RBCs suspension was adjusted to 10 g/dL using a Sysmex KX-21NV hematology analyser (Sysmex Co., Kobe, Japan). Then, CO gas (Kumamoto Sanso, Kumamoto, Japan) was gently bubbled through the RBCs suspension for 10 min.

### 5.6 Measurement of CO concentration in the liver

ND-fed ICR mice (male, 6 weeks old) and 4 weeks-MCDD-fed C57BL/6 N mice (male, 13 weeks old) were injected intravenously with CO-RBCs (1400 mg Hb/kg). To perfectly dissociate CO from Hb or hemoprotein, whole liver samples were mixed with saponin (5 mL; Nacalai Tesque) in a vial (10 mL) and incubated for 4 h. The concentration of the CO in the headspace of the vial was qualified by gas chromatography with a TRIlyzer mBA3000 (Taiyo Instruments, Inc., Osaka, Japan).

### 5.7 Cell culture and treatment

RAW264.7 and HepG2 cells were purchased from the RIKEN Bioresource Centre Cell Bank (Ibaraki, Japan). RAW264.7 cells were cultured in Dulbecco's modified Eagle medium (DMEM) high glucose (Wako) supplemented with 10 % fetal bovine serum (FBS; Hyclone Laboratories, Logan, UT), 100 U/ml penicillin (Invitrogen, Carlsbad, OH) and 100 μg/mL streptomycin (Invitrogen). HepG2 cells were grown in Roswell Park Memorial Institute 1640 (Sigma-Aldrich), containing 10 % FBS, 100 U/mL penicillin, and 100 μg/mL streptomycin. Cells were maintained at 37 °C in a humidified atmosphere with 5 % CO_2_. PA (Sigma-Aldrich) was dissolved in 50 % ethanol at 60 °C. The PA solution was mixed in defatted albumin solution in PBS (pH 7.4, 37 °C) to achieve a 7:1 M ratio and the solution was filtered through a 0.2-μm filter. The concentrations of the CO-RBCs and O_2_-RBCs solutions were determined considering the results shown in Fig. S5. Finally, hemoCD (Doshisha University, Kyoto, Japan) was used to confirm that the effect of CO-RBCs was due to CO.

### 5.8 Western blot analysis

Western blot analyses were performed as described previously [37]. Briefly, tissue or cell lysates were collected in RIPA buffer (1 M Tris-HCl, pH 7.5, 250 mM NaCl, 0.1 % Triton X, 1 % SDS) containing a 1 % phosphatase inhibitor cocktail (Nacalai Tesque) and a 1 % protease inhibitor cocktail (Nacalai Tesque). The lysates were loaded and then separated on a 10 % SDS-PAGE gel. The proteins were transferred to PVDF membranes (Millipore, Temecula, CA) and then incubated with primary antibodies dissolved in Tris buffered saline containing 0.1 % Tween 20 (TBS-T) overnight at 4 °C. The membranes were then incubated with secondary antibodies dissolved in TBS-T for 2 h at room temperature. Protein bands were visualised using ImageQuant™ LAS 4000 mini (GE Healthcare UK Ltd, Buckinghamshire, England), and the intensity of the protein bands was quantified using ImageJ software. The primary and secondary antibodies are detailed in Table S4.

### 5.9 Plasma biochemical parameters

Blood samples were taken from the inferior vena cava at 4 weeks after the beginning of MCDD feeding. Blood samples were centrifuged (1000×*g*, 10 min) to obtain plasma samples to analyse plasma biochemical parameters and hydroperoxide levels. Plasma triglyceride levels were analysed using Fuji DRI-CHEM 7000Z and DRI-CHEM slides (Fujifilm, Tokyo, Japan). Plasma AST and ALT levels were analysed by transaminase CII-test (Wako) according to the manufacturer's protocol.

### 5.10 Measurement of hepatic TG content

Hepatic TGs were extracted using the method described by Chikamatsu et al. [81]. After extraction, the TG concentration in the extract was determined using the Triglyceride E test (Wako).

### 5.11 Quantitative reverse transcription polymerase chain reaction (RT-PCR) analysis

Quantitative RT-PCR analyses were performed as described in our previous report [37]. In brief, total RNA was extracted using RNAiso PLUS (TaKaRa Bio Inc., Shiga, Japan). The concentration and purity of the RNA extract was determined by the absorbance at 260 and 280 nm. cDNA was synthesised using the Prime Script® RT master mix (TaKaRa Bio Inc.). Quantitative RT-PCR analysis was performed in an iCycler (Bio-Rad, Hercules, CA) with an iQ5 qRT-PCR detection system (Bio-Rad) using SYBR® PremixEx *Taq*II (TaKaRa Bio Inc.). The data were evaluated using the delta-delta Ct method, normalised to the expression of GAPDH. The sequences of the oligonucleotide primers are provided in Table S5.

### 5.12 Kupffer cell depletion

Upon initiating MCDD feeding, mice were injected intraperitoneally with GdCl_3_ (10 mg/kg; Sigma-Aldrich) to eliminate Kupffer cells. GdCl_3_ was then administered twice a week to prevent Kupffer cell repopulation.

### 5.13 *Measurement* of TNF-α, IL-1β and IL-10 concentration

TNF-α, IL-1β and IL-10 levels in plasma samples *in vivo* and in culture supernatants *in vitro* were determined by enzyme-linked immunosorbent assay (ELISA; Biolegend, San Diego, CA) according to the manufacturer's protocol.

### 5.14 Immunofluorescence

In immunofluorescence studies using mouse liver samples, paraffin sections of liver samples were heated with an antigen retrieval solution (HistoVT One; Nacalai Tesque) for 40 min and then incubated with a blocking reagent (Block Ace; DS Pharma Biomedical) for 1 h at room temperature. The liver samples were then incubated overnight at 4 °C with primary antibodies before exposure to secondary antibodies at room temperature for 90 min. The slides were observed under a fluorescence microscope (BZ-8000; Keyence). The primary and secondary antibodies used are provided in Table S6.

### 5.15 Quantification of malondialdehyde (MDA) in the liver

Frozen liver samples were ground into powder and homogenised in 1 mL of RIPA buffer containing 1 % protease inhibitor. The supernatant was collected by centrifugation (10,000×*g*, 10 min, 4 °C), and MDA levels in the solution were quantified using a BIOXYTECH MDA-586 kit (OxisResearch, Burlingame, CA).

### 5.16 Quantification of hydroperoxide in plasma

Murine blood samples were centrifuged (1000×*g*, 10 min, 4 °C) to isolate the plasma before analysis using Free Carpe Diem (Wismerll Company Ltd., Tokyo, Japan) to quantify plasma hydroperoxide.

### 5.17 Quantification of ATP in liver and HepG2 cells

Intracellular ATP levels were determined using an ATP assay kit (Toyo B Net, Tokyo, Japan). Briefly, HepG2 cells were seeded in 96-well plates which were then incubated overnight at 37 °C and treated with PA (100 μM) and/or CO-RBCs (0.5 mg Hb/ml) for 12 h at 37 °C. To extract ATP, the cells were incubated with ATP extraction reagents for 5 min at room temperature. In the case of tissues, livers were homogenised in sterile water, and the supernatant was collected after centrifugation (1000×*g*, 10 min, 4 °C) and diluted 10 times in RIPA buffer. The ATP solutions extracted from cells or tissues were reacted with ATP luminescent reagents and luminescence levels were measured using a luminometer (Lumat3 LB9508, Berthold Technologies, Bad Wildbad, Germany).

### 5.18 Measurement of lipid accumulation in HepG2 cells

HepG2 cells were seeded in 12-well plates, which were then incubated overnight at 37 °C and treated with PA (100 μM) and/or CO-RBCs (0.5 mg Hb/ml) or O_2_-RBCs (0.5 mg Hb/mL) for 24 h at 37 °C. After washing with PBS, the cells were fixed by incubating them in 10 % phosphate buffered formalin for 10 min at room temperature. Oil Red O solution was added and incubated for 20 min, then washed and observed for labelling under a microscope (BZ-8000). For quantification, 2-propanol (200 μL; Nacalai Tesque) was added to the cells after staining, incubated for 5 min, and the absorbance of the supernatant at 490 nm was measured.

### 5.19 Statistical analysis

Statistical analyses were performed with Excel (Microsoft) and Prism (GraphPad). Error bars represent the means ± standard error (S.E.). The means of two groups of data were compared by unpaired *t*-test. Two groups of data were compared by student's t-test. More than two groups were compared by one-way analysis one-way ANOVA analysis followed by Tukey's multiple comparisons. Data were considered statistically different at P < 0.05. P < 0.05 is indicated with single asterisks, P < 0.01 with double asterisks, P < 0.001 with triple asterisks, and P < 0.0001 with quadruple asterisks.

## CRediT authorship contribution statement

**Hiroki Yanagisawa:** Writing – original draft, Project administration, Investigation, Formal analysis, Data curation. **Hitoshi Maeda:** Writing – review & editing, Supervision, Investigation, Funding acquisition, Formal analysis. **Isamu Noguchi:** Writing – original draft, Visualization, Methodology, Investigation, Formal analysis, Data curation, Conceptualization. **Motohiko Tanaka:** Supervision, Project administration. **Naoki Wada:** Writing – original draft, Investigation, Formal analysis, Data curation. **Taisei Nagasaki:** Investigation. **Kazuki Kobayashi:** Investigation. **Gai Kanazawa:** Investigation. **Kazuaki Taguchi:** Supervision. **Victor Tuan Giam Chuang:** Writing – review & editing, Supervision. **Hiromi Sakai:** Supervision, Resources. **Hiroyuki Nakashima:** Methodology. **Manabu Kinoshita:** Supervision, Methodology. **Hiroaki Kitagishi:** Supervision, Resources. **Yasuko Iwakiri:** Writing – review & editing, Supervision. **Yutaka Sasaki:** Supervision. **Yasuhito Tanaka:** Supervision. **Masaki Otagiri:** Project administration. **Hiroshi Watanabe:** Writing – review & editing, Project administration. **Toru Maruyama:** Writing – review & editing, Project administration, Funding acquisition, Conceptualization.

## Declaration of competing interest

The authors declare that they have no known competing financial interests or personal relationships that could have appeared to influence the work reported in this paper.

## Data Availability

No data was used for the research described in the article.
