# Supplementary material for: Carbon monoxide-loaded red blood cells ameliorate metabolic dysfunction-associated steatohepatitis progression via enhancing AMP-activated protein kinase activity and inhibiting Kupffer cell activation
Source: Redox Biol. 2024 Aug 17;76:103314. doi: 10.1016/j.redox.2024.103314 (PMC11381851; doi:10.1016/j.redox.2024.103314)
Supplement: Multimedia component 1 [file mmc1.docx]

**Supplementary Material**

**
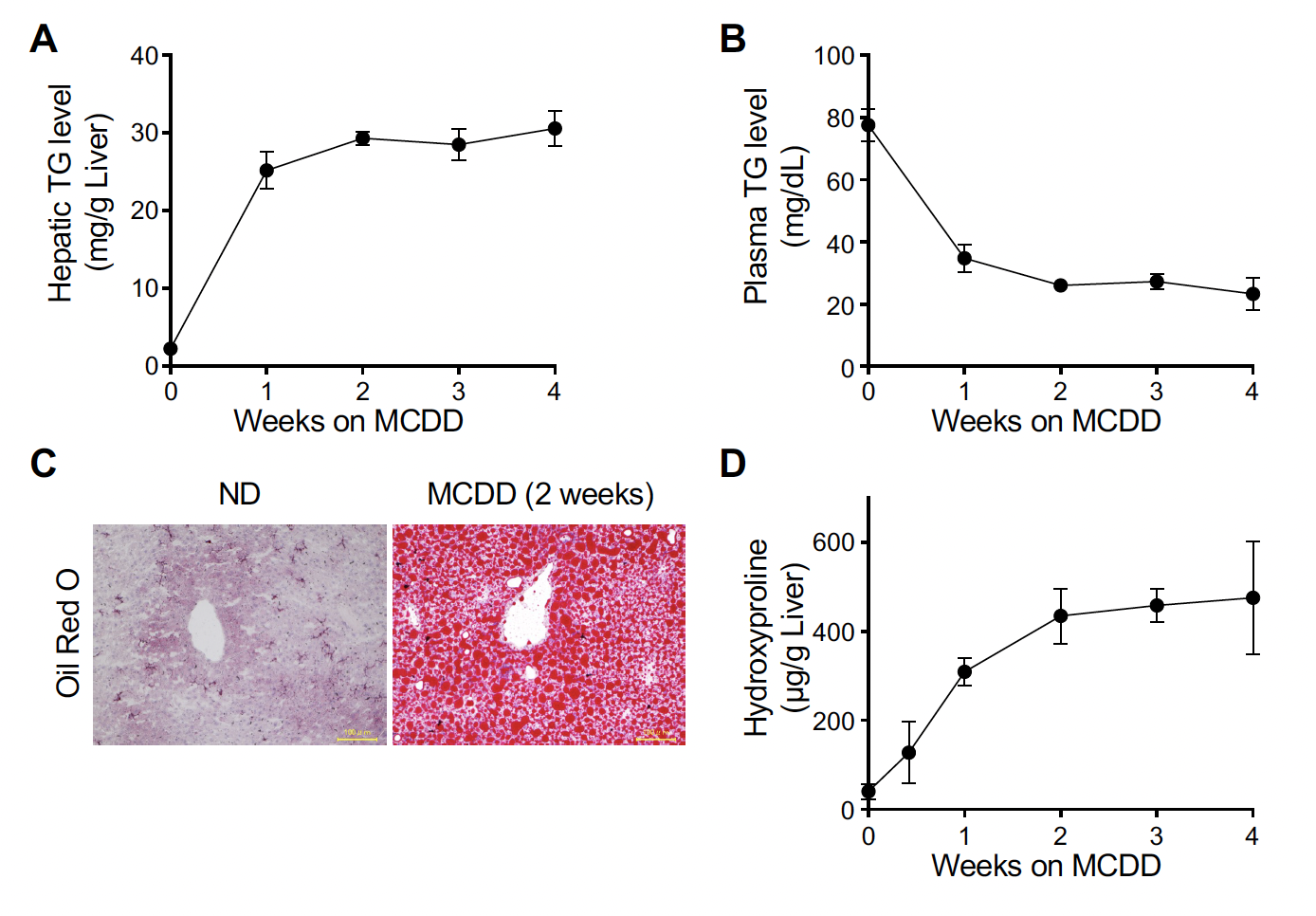
**

***S. Fig. 1 | Lipid accumulation and fibrosis status after MASH induction with MCDD feeding.*** (A) Hepatic and (B) plasma TG and (D) hydroxyproline levels were monitored over 4 weeks after the start of MCDD feeding (n = 3). (C) Representative photomicrographs of Oil Red O-stained liver sections at 2 weeks after the start of MCDD feeding (scale bars, 100 μm). Results are expressed as the means ± S.E.

**
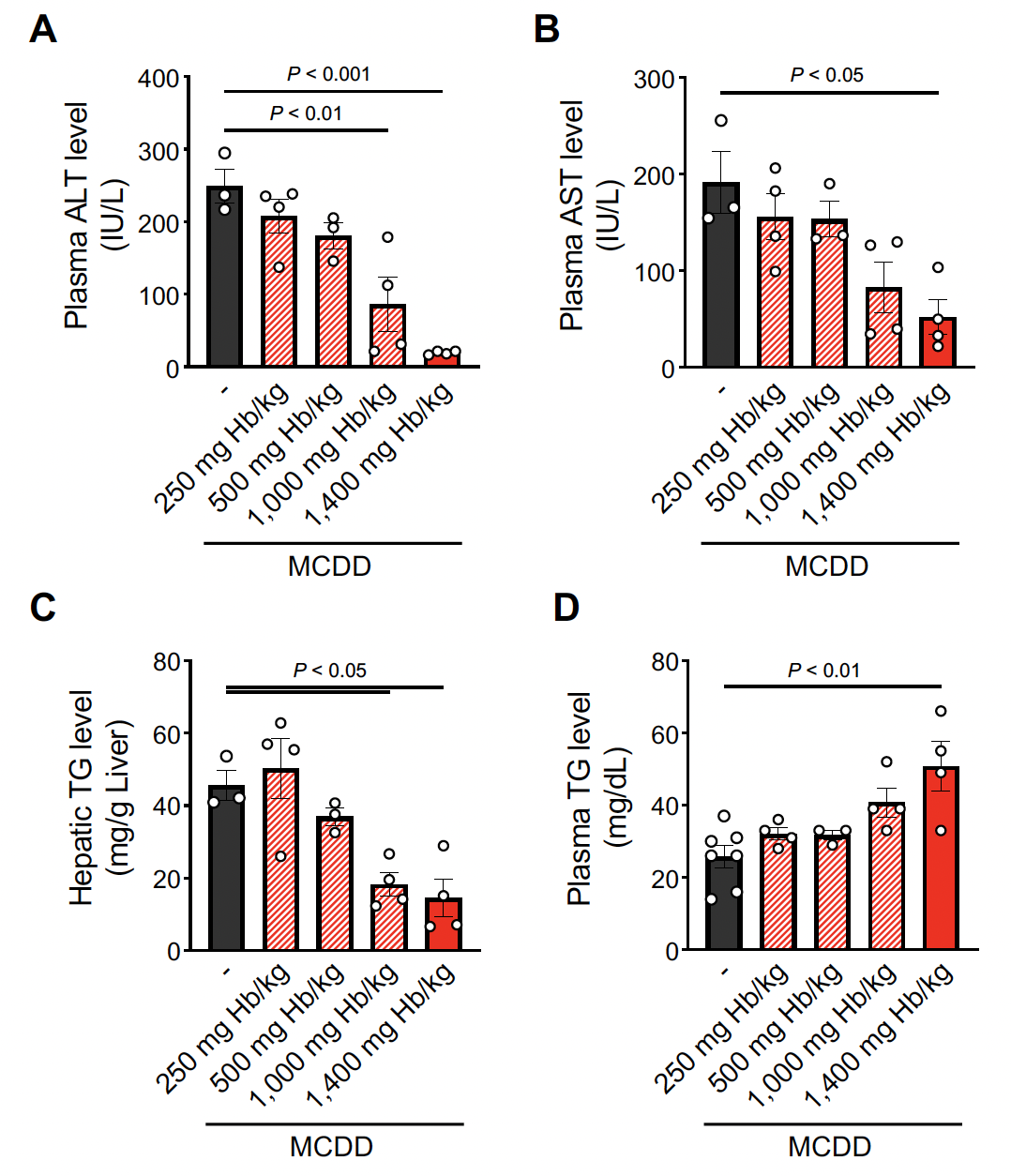
**

***S. Fig. 2 | Dose-dependent recovery effects of CO-RBCs on MCDD-fed MASH mice.*** Plasma (A) ALT and (B) AST levels, and (C) hepatic and (D) plasma TG levels were measured in MCDD-fed MASH mice, 2 weeks after the start of administration of each concentration of CO-RBCs (n = 3-4/group). Results are expressed as the means ± S.E.


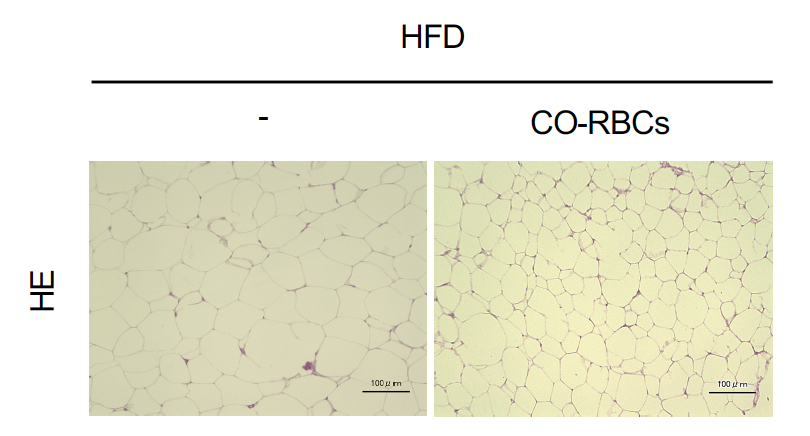


***S. Fig. 3 | Inhibition of adipocyte hypertrophy in HFD-fed MASH mice upon CO-RBCs treatment.*** Representative photomicrographs of hematoxylin and eosin (HE) stained epididymal white adipose tissue sections collected from HFD-fed MASH mice 2 weeks after the start of CO-RBCs administration (scale bars, 100 μm).

**
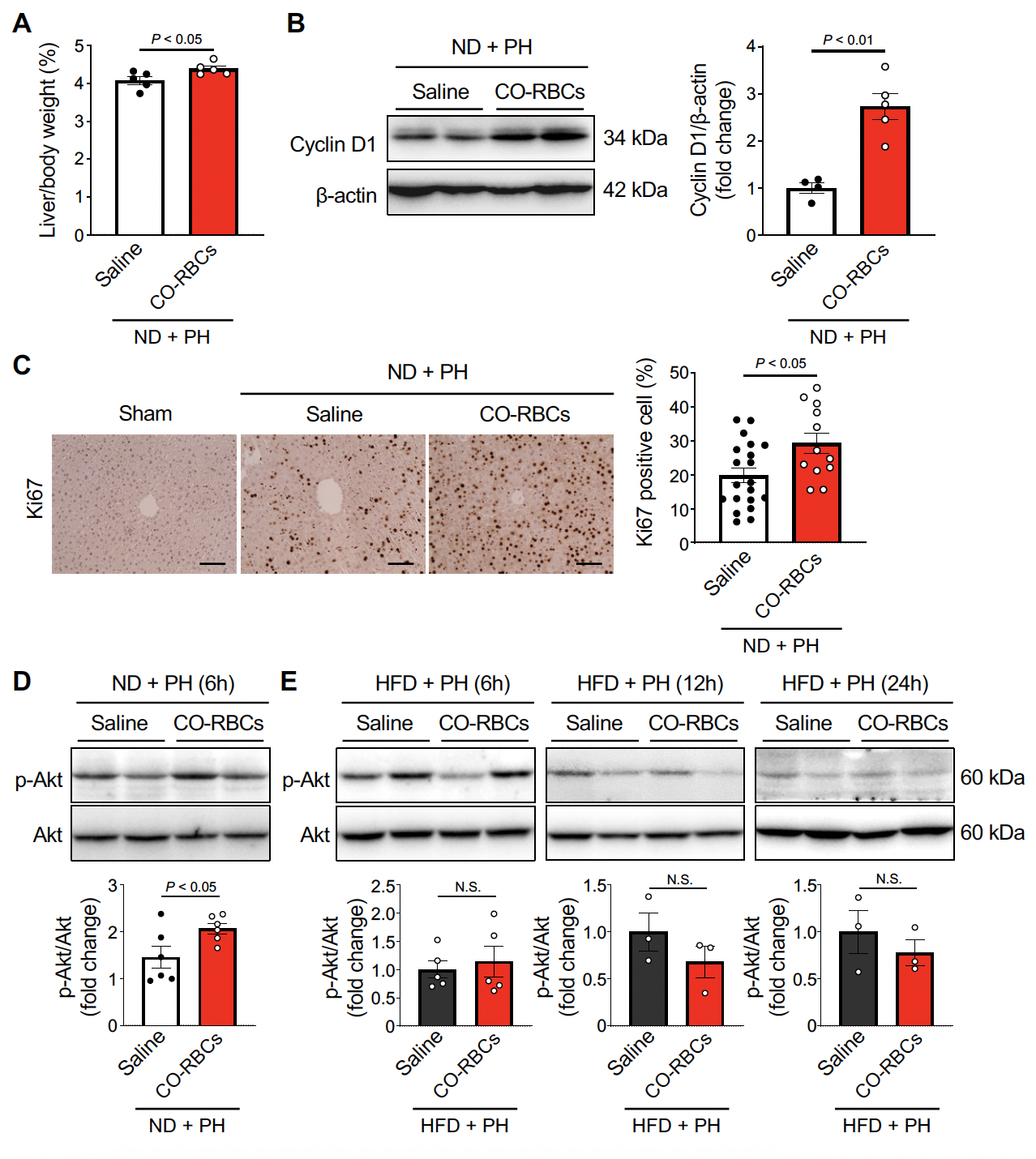
**

***S. Fig. 4 | Effects of CO-RBCs on liver regeneration and Akt levels in normal mice.*** (A) Liver weight as a percentage of body weight at 48 hours after 70% partial hepatectomy (PH) in normal diet (ND)-fed normal mice (n = 5/group). (B) Hepatic Cyclin D1 expression was determined by western blotting 48 hours after PH in ND-fed normal mice (n = 4-5/group). (C) Representative images of immunostaining for Ki67 in the liver 48 hours after PH or a sham treatment in ND-fed normal mice (left panel; scale bar, 100 μm) and the percentage of Ki67-positive hepatocytes (right panel; n = 12/group). (D) The Akt activity of ND-fed mice 6 hours after PH was measured using western blotting (upper panel) and quantified (lower panel; n = 6/group). (E) The Akt activity of high fat diet (HFD)-fed MASH mice 6, 12, and 24 hours after PH was measured using western blotting (upper panel) and quantified (lower panel; n = 3-5/group). Results are expressed as the means ± S.E; N.S., not significant.

**
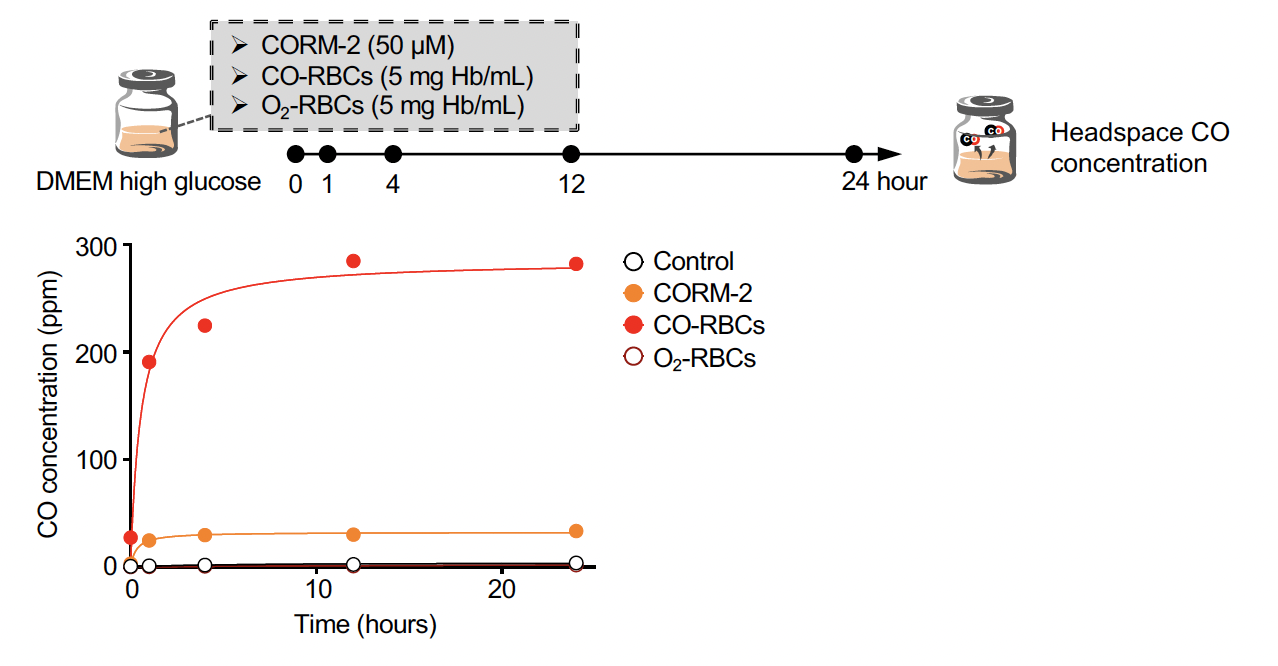
**

***S. Fig. 5 | Amount and rate of CO release by CO-RBCs in vitro.*** Schematic of the study protocol to evaluate the *in vitro* CO release by CO-RBCs compared with CORM-2. CORM-2 (50 μM), CO-RBCs (5 mg Hb/mL), and O_2_-RBCs (5 mg Hb/mL) were mixed with DMEM high glucose supplemented with 10% FBS, 100 U/mL penicillin, and 100 μg/mL streptomycin in a vial, and after each incubation, the CO concentration in the headspace was measured. The concentration of CO-RBCs (5 mg Hb/mL) was adjusted to the amount of CO loaded in CORM-2 (50 μM). As CO-RBCs released about 10 times as much CO as CORM-2 into the headspace under these conditions, a concentration of 0.5 mg Hb/mL of CO-RBCs was used for further experiments.

***Supplemental Table 1 | Physiological parameters of MCDD-fed MASH mice after a 4-week exposure to CO-RBCs or O_2_-RBCs.***

|  | ND | MCDD/- | MCDD  /CO-RBCs | MCDD  /O_2_-RBCs |
| --- | --- | --- | --- | --- |
| Body Weight (g) | 25.8 ± 0.9 | 16.2 ± 0.6 ^**^ | 17.3 ± 0.6 ^**^ | 17.1 ± 0.5 ^**^ |
| Liver Weight  (mg/g Body Weight) | 51.7 ± 4.3 | 40.1 ± 6.0 ^*^ | 44.4 ± 5.3 | 44.4 ± 3.9 ^*^ |
| Food Intake  (g/kg Body Weight) | 159.7 ± 12 | 162.1 ± 25.3 | 164.0 ± 28.1 | 178.9 ± 32.2 |

**P* < 0.05, ***P* < 0.01 vs. ND-fed group.

***Supplemental Table 2 | Physiological parameters of HFD fed MASH mice after a 2-week exposure to CO-RBCs.***

|  | HFD/- | HFD/CO-RBCs |
| --- | --- | --- |
| Body Weight (g) | 23.9 ± 0.7 | 23.5 ± 0.4 |
| Liver Weight  (mg/g Body Weight) | 45.2 ± 1.4 | 47.2 ± 1.6 ^*^ |
| Food Intake  (mg/g Body Weight) | 47.0 ± 1.0 | 47.5 ± 1.1 |

**P* < 0.05 vs. HFD/- group.

***Supplemental Table 3 | List of antibodies used in enzyme immunostaining.***

| Primary antibody | Company | Catalog # | Dilution rate |
| --- | --- | --- | --- |
| Human HO-1 | Bethyl Laboratories | A303-662A | 1:100 |
| Mouse HO-1 | Enzo Life Science | ADI-SPA-896 | 1:100 |
| Mouse 4-HNE | Bioss | bs-6313R | 1:50 |

***Supplemental Table 4 | List of antibodies used for western blot analyses.***

| Primary antibody | Company | Catalog # | Dilution rate |
| --- | --- | --- | --- |
| HO-1 | Enzo Life Science | ADI-SPA-896 | 1:2,000 |
| TLR4 | Invitrogen | 14-9917-82 | 1:1,000 |
| Phospho-AMPKα | Cell Signaling Technology | 2535 | 1:1,000 |
| AMPKα | Cell Signaling Technology | 5832 | 1:1,000 |
| Phospho-Akt (Ser473) | Cell Signaling Technology | 4058 | 1:1,000 |
| Akt | Cell Signaling Technology | 9272 | 1:1,000 |
| CyclinD1 | Abcam | ab134175 | 1:10,000 |
| β-actin | Sigma-Aldrich | A5411 | 1:5,000 |
| Secondary antibody | Company | Catalog # | Dilution rate |
| anti-rabbit IgG-HRP | Santa Cruz Biotechnology | sc-2357 | 1:10,000 |
| anti-mouse IgG-HRP | Santa Cruz Biotechnology | sc-516102 | 1:10,000 |

***Supplemental Table 5 | List of primers used for quantitative real-time polymerase chain reaction.***

| Target gene | Forward primer (5’→3’) | Reverse primer (5’→3’) |
| --- | --- | --- |
| Mouse α-SMA | AGCCATCTTTCATTGGGATGG | CCCTGACAGGACGTTGTTA |
| Mouse TLR4 | TTCAGAGCCGTTGGTGTATC | CCCATTCCAGGTAGGTGTTT |
| Mouse PPAR-α | TTTCGGCGAACTATTCGGCTG | TTTGTGGATCCGGCAGTTAAGA |
| Mouse PPAR-γ | CCACCAACTTCGGAATCAGCT | TTTGTGGATCCGGCAGTTAAC |
| Mouse PGC-1α | TCTCAGTAAGGGGCTGGTTG | AGCAGCACACTCTATGTCACTC |
| Mouse GAPDH | AACTTTGGCATTGTGGAAGG | ACACATTGGGGGTAGGAACA |

***Supplemental Table 6 | List of antibodies used for immunofluorescence analyses.***

| Primary antibody | Company | Catalog # | Dilution rate |
| --- | --- | --- | --- |
| α-SMA | Abcam | ab5694 | 1:100 |
| TLR4 | Invitrogen | 14-9917-82 | 1:100 |
| NO_2_-Tyr | Merk-Millipore | AB5411 | 1:100 |
| 8-OHdG | Santa Cruz Biotechnology | sc-66036 | 1:100 |
| Secondary antibody | Company | Catalog # | Dilution rate |
| Alexa Fluor 546 goat  anti-rabbit IgG | Invitrogen | A-11010 | 1:200 |
| Alexa Fluor 647 donkey  anti-goat IgG | Invitrogen | A-21447 | 1:200 |
